# Operative treatment of nonunions in the elderly: Clinical and radiographic outcomes in patients at minimum 75 years of age

**DOI:** 10.1186/s12877-022-03670-8

**Published:** 2022-12-20

**Authors:** Clinton H. van Trikt, Johanna C. E. Donders, Craig E. Klinger, David S. Wellman, David L. Helfet, Peter Kloen

**Affiliations:** 1 Department of Orthopedic Surgery and Sports Medicine, Amsterdam University Medical Center, Amsterdam Movement Sciences, Amsterdam, The Netherlands; 2 Orthopaedic Trauma Service, Hospital for Special Surgery and New York Presbyterian Hospital, Weill Cornell Medicine, New York, NY USA; 3 Orthopaedic Trauma Service, Westchester Medical Center, New York Medical College, Valhalla, NY USA

**Keywords:** Elderly, Operative management, Geriatric trauma, Nonunions, Non-union scoring system, Humerus, Femur, Femur periprosthetic

## Abstract

**Background:**

Limited information exists on nonunion treatment in the elderly. This retrospective study evaluates whether results of operative treatment of nonunion of the humerus or femur in patients aged ≥ 75 years are comparable to those in younger patients.

**Methods:**

We identified patients age ≥ 75 years with a nonunion of humerus or femur treated with open reduction and internal fixation. The Non-Union Scoring System was calculated. Complications, clinical outcome, and radiographic findings were assessed. Primary endpoint was nonunion healing. A literature review compared time to healing of humeral and femoral nonunion in younger populations.

**Results:**

We identified 45 patients treated for a nonunion of humerus or femur with > 12 months follow-up. Median age was 79 years (range 75–96). Median time to presentation was 12 months (range 4–127) after injury, median number of prior surgeries was 1 (range 0–4). Union rate was 100%, with median time to union 6 months (range 2–42). Six patients underwent revision for persistent nonunion and healed without further complications.

**Conclusions:**

Using a protocol of debridement, alignment, compression, stable fixation, bone grafting and early motion, patients aged 75 years or older can reliably achieve healing when faced with a nonunion of the humerus or femur.

**Level of Evidence:** IV.

## Background

Musculoskeletal problems in the elderly are seldom fatal, but quality of life and function is often severely affected after impaired fracture healing. Fortunately, bone has intrinsic capacity to heal after a fracture [1]. This reparative (almost regenerative) potential is surprisingly well maintained throughout life [2]. The complex but well-orchestrated events seen after a fracture closely resemble bone development during embryogenesis and, in the majority of cases, leads to complete healing and restoration of pre-fracture mobility and function [3]. A small subset of patients will develop a nonunion [4]. To complicate matters, there is no consensus as to what defines a nonunion. One pragmatic definition is “a fracture that will not heal without surgical intervention” [5].

Risk factors for development of a nonunion are numerous. There are fracture-related factors (e.g., open injuries, and devitalized segments), patient-related factors (ex. nutrition, comorbidities, and smoking), and medication factors. Additionally, there are surgeon-related factors such as surgical technique (ex. biologically friendly versus extensile dissections), mechanical stability provided, and quality of reduction [6]. Finally, infection can play a significant role, especially if the nonunion presents after attempted surgical intervention.

Whether age is an independent risk factor for nonunion remains unclear. There is evidence that bone repair is delayed in the elderly compared to younger patients [7–9]. Recent studies (animal and human) elucidated small but potentially important differences between fracture healing in these two age groups [9–11]. With increased aging, there are less mesenchymal stem cells (MSC) or osteoprogenitor cells [7, 8, 11–13]. Stem cells from elderly patients produce less bone than those from younger individuals [14]. MSCs in the elderly differentiate more into adipocytes rather than osteoblasts [15]. Additionally, recent studies suggest an increased inflammatory state in the elderly, known as *inflamm-aging,* triggers skeletal stem/progenitor cell dysfunction and cell senescence [12, 13]. Despite progress made during recent decades in fracture treatment, the overall incidence of non-unions remains 2–5% for all fracture types [16, 17]. Higher incidence of failure in nonunion surgery is expected in elderly given their delayed bone repair. Furthermore, the number of elderly patients requiring nonunion treatment will grow due to rising life expectancy [18, 19].

In most patients, a nonunion is a disabling condition resulting in pain, reduced activity levels, and inability to return to the pre-injury functional status [20]. A nonunion may even have a higher impact upon the elderly population, who often have compromised self-reliance and mobility, and lead to increased need for long-term nursing homecare, decreased quality of life, social isolation, depression, and decreased self-confidence [21].

The goal of nonunion surgery in the elderly is achieving the highest functional recovery. This can be accomplished with prosthetic replacement in some cases. Prosthetic replacement has a role in nonunion of the femoral neck, distal femur, as well as in the proximal humerus and distal humerus. However, most nonunions will require formal open reduction and internal fixation (ORIF) and potentially bone grafting [22, 23]. There are many challenging issues for any type of ORIF in the elderly, which often include a combination of decreased soft tissue quality, prior scar tissue, poor bone stock, and joint stiffness of the affected limb. The same age-related comorbidities that compromise fracture healing (e.g., diabetes, malnutrition, osteoporosis, osteopenia, and polypharmacy including chronic opioid use) increase the complexity level of operative fracture treatment and after-care in the elderly [7, 9, 18, 24].

Although many studies have reported on patient outcomes following nonunion treatment, to date no studies have reported outcomes of nonunion treatment in patients aged 75 years or older [25, 26]. Specifically, we were interested in whether treatment of nonunions of the humerus and femur resulted in similar osseus union rates and time to union in the elderly as compared to younger cohorts reported in the literature. Thus, the first purpose of this study was to assess outcomes of operative nonunion treatment in patients aged 75 years or older. We assessed the rate of radiographic and clinical time to osseous union, subsequent surgeries, and complications. We then compared our results with findings from the literature.

## Methods

Institutional Review Board approval was obtained for this retrospective research study. All patients aged 75 years or older with an operatively treated nonunion of the humerus or femur between 1996–2019 were identified in two tertiary referral centers (Hospital for Special Surgery and Amsterdam Medical Center). Other nonunion locations were excluded (*n* = 15). All patients were treated by three fellowship-trained orthopedic trauma surgeons on this study (DLH, PK, and DSW). Exclusion criteria consisted of pathologic fractures and follow-up less than 12 months (*n* = 30). All data were prospectively recorded in orthopedic trauma databases. The fracture leading to the nonunion was classified according to the AO/OTA classification (including the periprosthetic classification) [27]. Nonunion type was classified according to Weber and Çech and the Non-Union Scoring System (NUSS) according to Calori et al. which combines nonunion type and previous treatment with patient characteristics (e.g., ASA-score, comorbidity, medication, blood tests) [28, 29]. The NUSS ranges from 0–100 points, with 0–25 points representing a straightforward nonunion. A score of 75–100 indicates the need for amputation, arthrodesis or prosthetic replacement [30, 31]. Radiographic assessment included presence or absence of bone healing (cortical bridging of at least 3 cortices) using standard radiographs and/or CT-scan [32]. Subsequent revision surgery to achieve bony union and post-operative complications were also documented. Patients were seen at regular intervals (6 weeks, 3, 6, 12 months). Clinical outcome was based on pain and ability to weight-bear (for femur nonunion). Patient demographics are listed in Table 1.

**Table 1:** Demographic patient data

Demographic data	Total	Humerus	Femur
**Number of patients**	45	16 (35.6%)	29 (64.4%)
**Distribution M: F**	13: 32	3: 13	10: 19
**Median age in years (range)**	79 (75 – 96)	78.5 (75 – 95)	80 (75 – 96)
**Median NUSS (range)**	32 (17 – 48)	33 (17 – 46)	32 (24 – 48)
**Mechanism of injury**	Fall: 42 (93.3%)	Fall: 15 (93.8%)	Fall: 27 (93.1%)
	MVA: 3 (6.7%)	MVA: 1 (6.3%)	MVA: 2 (6.9%)
**Median number of previous surgeries (range)**	1 (0 – 4)	0 (0 – 4)	1 (1 – 4)
**ASA**	1: 1 (2.2%)	2: 11 (68.8%)	1: 1 (3.5%)
	2: 28 (62.2%)	3: 5 (31.3%)	2: 17 (58.6%)
	3: 16 (35.6%)		3: 11 (37.9%)
**Fracture type according to the AO/OTA classification**	A1: 4 (8.9%)	A1: 3 (18.8%)	A1: 1 (3.5%)
	A2: 7 (15.6%)	A2: 4 (25%)	A2: 3 (10.3%)
	A3: 8 (17.8%)	B1: 1 (6.3%)	A3: 8 (27.6%)
	B1: 1 (2.2%)	B2: 3 (18.8%)	B2: 1 (3.5%)
	B2: 4 (8.9%)	B3: 1 (6.35%)	B3: 1 (3.5%)
	B3: 2 (4.4%)	C2: 2 (12.5%)	C3: 1 (3.5%)
	C2: 2 (4.4%)	C3: 2 (12.5%)	
	C3: 3 (6.7%)		
	Periprosthetic		Periprosthetic
	A: 1 (2.2%)		A: 1 (3.5%)
	B: 1 (2.2%)		B: 1 (3.5%)
	C: 11 (24.4%)		C: 11 (37.9%)
	D: 1 (2.2%)		D: 1 (3.5%)
**Weber & Çech nonunion classification**	Atrophic: 14 (31.1%)	Atrophic: 9 (56.3%)	Atrophic: 5 (17.2%)
	Oligotrophic: 23 (51.1%)	Oligotrophic: 7 (43.8%)	Oligotrophic: 16 (55.2%)
	Hypertrophic: 8 (17.8%)		Hypertrophic: 8 (27.6%)

## Surgical technique

The type of surgery was at the discretion of the treating surgeon involved in their care. Two of the three treating surgeons in this study were fellowship trained by the third. Given this training background, there were only minor differences in surgical tactics and choice of fixation. All surgeries included infection screening using tissue sampling before administration of intravenous antibiotics (2gm cephalosporin). For distal and shaft nonunion, a tourniquet was used at 250 mm Hg up to a maximum of 2 h. Whenever possible, the previous scar tissue was utilized for the surgical approach. After exposure, all failed or loose hardware was removed. For oligotrophic and atrophic nonunions, intervening soft tissues were removed with a combination of scalpel, curette or ronguer. The medullary canal was drilled open, or the sclerotic end cap was removed with an oscillating saw. Debridement progressed until healthy bleeding bone was visible at both nonunion ends. For well-aligned hypertrophic nonunion, the intervening tissue of the nonunion was left intact. For distal femur and distal humerus nonunion with limited motion due to contracture and/or adhesions, a formal soft tissue release was performed [23, 33]. Reduction and temporary fixation was provided by K-wires, reduction clamps, and/or small plates. Depending on nonunion type and location and prior fixation, definitive fixation consisted of compression plating or nailing. When plating was selected, the surgical strategy consisted of, whenever possible, relatively long plates with hybrid fixation and compression across the nonunion site with rigid fixation [34]. Plating was performed in all humeral nonunions. We strongly prefer plating, as results of intramedullary nails for upper extremity long bone nonunions are potentially inferior [35, 36] For midshaft femur nonunion, either plates or nails were selected depending on nonunion type and surgeon preference. For nonunion in the meta-diaphyseal area of the femur, plating was performed.

Bone graft utilization was dependent on nonunion type. In most oligotrophic nonunions, and in all atrophic nonunions, bone graft was utilized. Iliac crest bone graft (ICBG) was used throughout the study period, as was demineralized bone matrix (DBM, Johnson&Johnson/DePuy/Synthes, Paoli, PA, and Osteotech, Eatontown, NJ). Bone morphogenetic proteins (BMP-2; Infuse, Medtronic Minneapolis, MN) was used in isolated cases. For large defects, fibula allograft or tricortical ICBG were used for structural support.

## Literature search

**Fig. 1:**
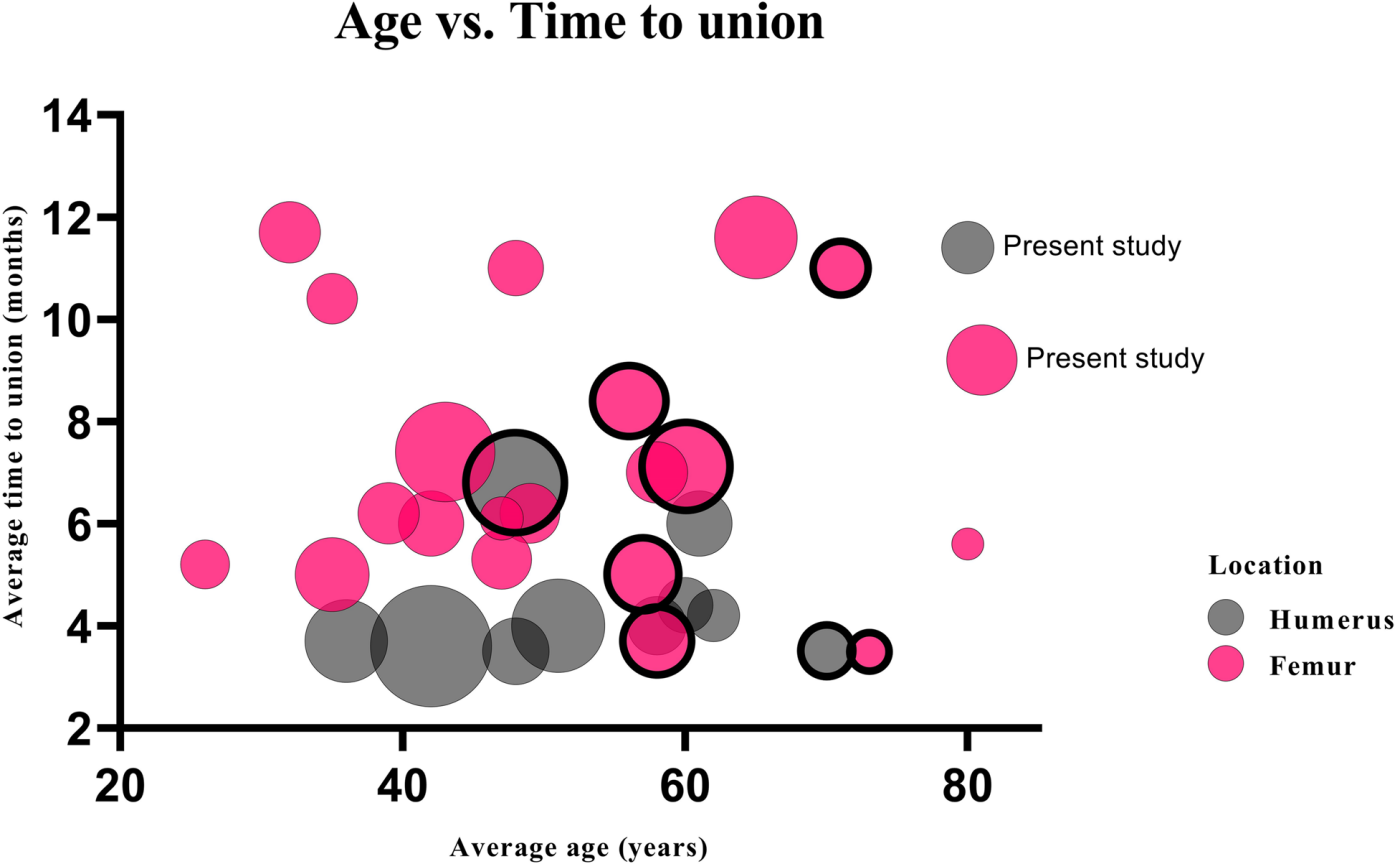
Scatterplot of our literature search (Table 4), with weighted markers based on size of the studies. The markers labeled with “present study” are the elderly patients from our study. The thick bordered markers are studies published previously by us

A literature search was completed (PubMed/Google Scholar) using search terms “nonunion”, “non-union”, “delayed union”, “femur”, “humerus”. Selection criteria were: i) time to union being measured, ii) a minimum of 6 patients, iii) age provided, and iv) English language. We compared our results with published findings in younger patients. A multi-variate regression analysis (corrected for sample size) was run to predict time to union based on age and location (Fig. 1).

## Results

**Fig. 2:**
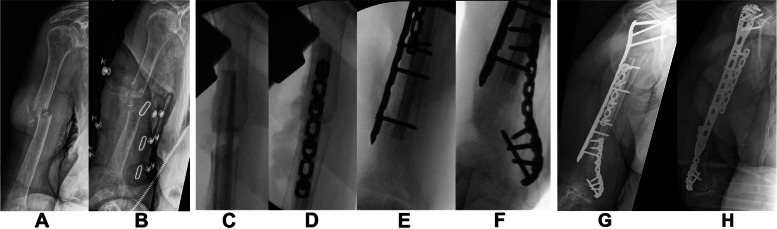
A 95-year-old female fell and sustained a midshaft humerus fracture which was treated with a Sarmiento brace at a local hospital. She was referred at five months for a second opinion and presented with pain, a flail arm and radiographic imaging demonstrating lack of fracture callus (**A**, **B**). Open reduction and internal fixation was performed with debridement of the nonunion site, removal of bone edges along the nonunion site, and intrameduallary placement of fibular allograft (**C**). This was followed by compression of the construct around the allograft with placement of an anterior 3.5 mm locking compression pelvic reconstruction plate anterolaterally and a long Philos plate laterally including screws through all 4 cortices through the allograft (**D**, **E**). During fluoroscopy at the end of surgical procedure the arm was externally rotated for a final image, which unfortunately led to an iatrogenic fracture of the metaphyseal distal humerus which was then fixated with a second contoured 3.5 mm pelvic recon locking compression pelvic reconstruction plate (**F**). She returned for routine follow-up and at 4 months radiographic and clinical findings demonstrated a healed humeral nonunion and fracture (**G**, **H**); and at most recent contact she is doing well, 99 years of age and has a pain-free arm

**Fig. 3:**
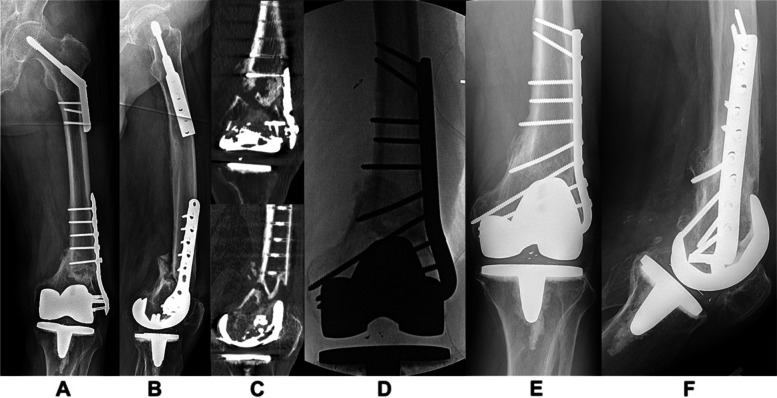
An 85-year-old male fell approximately 8.5 years following a left-sided total knee arthroplasty (and was also 9 years status-post left-sided hip fracture surgery). He sustained a supracondylar periprosthetic distal femur fracture and was surgically treated at a local hospital with a distal femur locking compression plate (LCP). He developed a nonunion and subsequently was treated with a bone stimulator. He presented to our institution 14 months following surgery. Anteroposterior (AP) and lateral imaging revealed a distal femur nonunion in varus deformity with posterior translation a flexion deformity (**A**,** B**). CT scan imaging further demonstrated the nonunion morphology (**C**). Revision open reduction and internal fixation (ORIF) was performed with LCP and screw removal, debridement of the nonunion site, (five) cultures taken, correction of deformity with placement of a 95^0^ blade plate and screws, demineralized bone matrix and bone morphogenic protein-2 (**D**). He returned for routine follow-up and healed 10 months following ORIF. Most recent follow-up AP and lateral radiographs at 20 months (**E**, **F**) revealed a healed nonunion in acceptable alignment and with maintenance of fixation and resumed his activities of daily living

Forty-five patients (consisting of 32 women and 13 men) with a humeral or femoral nonunion and aged 75 years or older were identified (Table 1). Median age at time of the index surgery was 79 years (range 75–96). Median age at time of injury was 78 years (range 72–94). Mechanism of injury included a fall in 42 patients (93.3%) and a motor vehicle accident in three (6.7%). In one patient (2.2%), the fracture was open (Gustilo Type 3A). Twenty-nine patients (64.4%) had at least one major medical co-morbidity and there were 13 smokers (28.9%). Median ASA-score was 2 (range 1–3). There were 16 humeral nonunions (35.6%) and 29 femoral nonunions (64.4%). Almost half of the 29 femur nonunions were periprosthetic (48.3%). None of the prostheses evidenced loosening. The NUSS had a median score of 32 (range 17–48). Fourteen (31.1%) of nonunions were atrophic, twenty-three (51.1%) were oligotrophic and eight (17.8%) were hypertrophic. All nonunions were extra-articular. After initial injury, 36 patients (80%) underwent ORIF, whereas 9 (20%) were managed non-operatively. Fifteen patients (33.3%) had undergone one or more revisions before the index operation (median 1, range 1–4). Median follow-up was 46.4 months (range 12–143). The index surgery was performed at a median of 12 months after initial injury (range 4–127). When plating was selected for femur nonunion surgery, a 95° blade plates was used in 53.6% (^**15**^/_**28**_**)** of patients (Johnson & Johnson/DePuy Synthes, West Chester, PA). Other cases were treated with anatomic variable angle locking plates (VA-LCP; Johnson & Johnson/DePuy Synthes), or 4.5 mm locking plates (Johnson & Johnson/DePuy Synthes). Double plating (parallel or orthogonal) was often employed. In two femoral shaft nonunions, an intramedullary device was used. Bone graft was frequently added (91.1%). Bone graft type varied between surgeons and changed over time. In seven patients (15.6%), only ICBG was added. In 13 patients (28.9%), DBM was used. In 14 patients (31.1%), a combination of ICBG and DBM was used. In 4 cases (8.9%), an intramedullary fibula allograft (FA) was used to augment bone stock. In 6 patients (13.3%), BMP-2 was added. Two representative cases are shown in Figs. 2 and 3.

## Outcomes

After the index surgery, 39 patients (86.7%) achieved osseous union at median time of 6 months (range 2–37); (Tables 2 and 3). Median time to union was longer in the humerus (6 months) than the femur (5 months). This may be explained by the characteristics of the humerus nonunion cohort, classified as either atrophic (56%) or oligotrophic (44%), as compared to the femoral nonunion group where 28% represented a hypertrophic nonunion.

**Table 2:** All patient data

Case	MOI	Open fracture (Gustilo)	Age / Sex	Major medical co-morbidity	Location	AO/OTA classification	Previous treatment(s) – Fixation type	Elapsed time since injury (months)	NU type (Weber-Çech)	NUSS
1	Fall		75F		Hu prox	11A2		7	AT	26
2	Fall		77F	DM	Hu prox	11C3		6	OT	34
3	Fall		80F	CVA	Hu prox	11A2		6	OT	36
4	Fall		84F	CVA, HTN	Hu shft	12A2	1 – Plate	5	AT	36
5	Fall		79F	AVR, CVA	Hu shft	12B2	1 – IMN	16	AT	30
6	Fall		75M		Hu shft	12A2		30	AT	34
7	Fall		75F	CVA, Spinal stenosis	Hu shft	12C3		7	AT	38
8	Fall		95F		Hu shft	12B3		15	OT	24
9	Fall		84F	Lymphoma	Hu shft	12A1	4 – Plate; Double-plate; ROH; Plate	32	OT	36
10	Fall		75F		Hu shft	12B2		4	AT	22
11	Fall		83M		Hu shft	12B1	1 – Plate	9	OT	24
12	Fall		85M		Hu shft	12A1		30	AT	28
13	Fall		75F	RA	Hu shft	12B2		8	AT	32
14	Fall		81F	DM	Hu shft	12A1	1 – IMN, cerclage wires	19	AT	34
15	Fall		78F	Cervical stenosis, Hysterectomy	Hu shft	12C2	2 – IMN; Plate	15	OT	17
16	MVA	IIIa	76F	CVA, DVT, HTN	Hu dist	13C2	1 – Plate	11	OT	46
17	Fall		82F	DVT, HTN, Osteoporosis	Fe prox	31A1	1 – IMN	12	AT	44
18	Fall		78F		Fe prox	31A3	2 – DHS, cerclage wires; Plate	11	OT	34
19	Fall		79F		Fe prox	31A3	1 – IMN	11	OT	32
20	Fall		80M	BPH, DM, Hypercholesterolemia	Fe prox	31A3	1 – IMN	12	OT	36
21	Fall		79M	PE, Poliomyelitis	Fe prox	31A3	1 – IMN	8	OT	38
22	Fall		83F	DM, HTN, Hypothyroidism	Fe prox	31A2	1 – IMN, cerclage wires	10	OT	28
23	Fall		80M		Fe prox	31A3	2 – IMN; Nail dynamization	13	HT	28
24	Fall		85F		Fe prox	31A2	1 – IMN	14	OT	28
25	Fall		75M		Fe prox	31A3	2 – IMN; Nail dynamization	14	AT	28
26	Fall		85F	MVR	Fe prox	31A3	1 – IMN	22	OT	34
27	Fall		78F	Cholecystectomy, Hepatitis, Pancreatitis, Splenectomy	Fe prox*	IV.3A	3 – Cerclage wires; Cerclage wires; Plate	13	OT	42
28	Fall		77M	BPH, Hyperlipidemia, HTN, Obesity	Fe prox*	IV.3C	1 – Plate, cerclage wires	5	OT	44
29	Fall		75M	BPH, HTN	Fe prox*	IV.3C	3 – Plate; Plate; Plate	34	OT	30
30	Fall		75F		Fe prox*	IV.3C	4 – NA; Plate; Plate; Plate	19	HT	36
31	Fall		76F		Fe prox*	IV.3C	1 – NA	4	OT	30
32	Fall		80F	Hypothyroidism	Fe prox*	IV.3C	2 – NA; Plate	20	HT	24
33	Fall		79F	AVS, Hypercholesterolemia	Fe prox*	IV.3C	2 – Ex fix; Ex fix	10	AT	32
34	Fall		86F		Fe shft	32B2	1 – DHS, plate	11	AT	36
35	MVA		96M		Fe shft	32A2	3 – NA; IMN; Plate	127	OT	36
36	Fall		81F	CVA	Fe shft	32B3	1 – IMN, cerclage wires	8	HT	26
37	Fall		81M	DM, RA	Fe shft	32C3	2 – Plate; Cerclage wires	22	HT	40
38	Fall		76F	DVT	Fe dist	33A3	4 – Plate; Plate; Plate; Plate	20	OT	48
39	Fall		77F	DM	Fe dist*	V.3C	1 – Plate	7	OT	32
40	MVA		78F	HPB	Fe dist*	V.3C	2 – Plate; Plate	12	OT	28
41	Fall		81F	AVR	Fe dist*	V.3C	1 – Plate	7	HT	28
42	Fall		77F	Asthma, HTN, RA	Fe dist*	V.3C	1 – Plate	13	OT	40
43	Fall		85M	CVA, Hepatitis, Hypothyroidism, MVR	Fe dist*	V.3C	1 – Plate	15	HT	26
44	Fall		85M		Fe dist*	V.3D	2 – Double-plate; Plate	13	HT	26
45	Fall		87F	Breast cancer, HTN	Fe dist*	V.3B	1 – Plate	7	AT	44
Case	**Fixation at Index surgery**			**Adjuvants**	**Months to union**	**Follow-up after last surgery (months)**	**Intra-operative cultures**	**Additional performed surgeries / Complications**		
1	1/3 tubular plate, cerclage wires			ICBG	34	86		ROH		
2	Philos plate, cerclage wires			ICBG + DBM	6	51		ROH (partial)		
3	Cerclage wires			ICBG + DBM	6	45		Nonunion healed, but AVN humeral head		
4	Double-plate (4.5 LCP (hybrid), 3.5 pelvic reconstruction (non-locking)			DBM	37	56				
5	Philos plate (locking)			DBM	13	13				
6	Double-plate (4.5 LCP (locking), 3.5 pelvic reconstruction LCP (standard))			ICBG + DBM	7	93				
7	Blade plate			ICBG	2	75				
8	Double-plate (4.5 LCP (hybrid), 3.5 pelvic reconstruction LCP (standard))			ICBG + DBM + FA	2	17				
9	Double-plate (3.5 LCP (hybrid), 3.5 pelvic reconstruction (standard))			ICBG + DBM	8	28				
10	Double-plate (3.5 LCP (hybrid), 3.5 pelvic reconstruction(hybrid))			DBM	3	13				
11	Double-plate (4.5 LCP (hybrid), 3.5 pelvic reconstruction (non-locking))			DBM	3	12				
12	Double-plate (4.5 LCP (hybrid), 3.5 pelvic reconstruction (non-locking))			DBM	17	34				
13	Double-plate (3.5 LCP dual plating, one straight (hybrid), one pelvic reconstruction (hybrid))			DBM	33	33				
14	4.5 LCP (hybrid)				5	69		Infection, radial nerve palsy and hardware failure: Revised at 2 months after index surgery with a staged procedure consisting of debridement, revision fixation, antibiotics and Osteoset beads (first stage) and addition of graft and 3.5 pelvic reconstruction LCP (hybrid) (second stage). Finally healed at 4 months after revision		
15	Double-plate (3.5 distal humerus metadiaphyseal plate (hybrid), 3.5 pelvic reconstruction (non-locking))			ICBG + DBM	2	12				
16	Double-plate (2 × 3.5 distal humerus plate (standard))			DBM	5	41	C. acnes			
17	Blade plate			DBM + BMP-2	11	21	CoNS	Persistent nonunion: Revised at 6 months after index surgery with double-plate (blade plate, 3.5 pelvic reconstruction plate). Finally healed at 5 months after revision		
18	Blade plate			ICBG	44	56		Persistent nonunion: Revised at 13 months after index surgery with blade plate. Finally healed at 42 months after revision		
19	Blade plate			DBM	3	14				
20	Blade plate			ICBG	25	143		Persistent nonunion: Revised at 7 months after index surgery with blade plate. Finally healed at 8 months after revision		
21	Blade plate			ICBG	7	58				
22	Blade plate			ICBG + DBM	9	78				
23	Blade plate			DBM	10	83				
24	Blade plate			ICBG	2	59				
25	Blade plate				2	43				
26	Blade plate			ICBG + DBM	3	14		ROH (partial)		
27	Cerclage wires			ICBG + DBM	3	85				
28	Double-plate (4.5 LCP (hybrid), 3.5 pelvic reconstruction (non-locking))			DBM + BMP-2	6	52				
29	VA distal femur LCP (hybrid)			ICBG + DBM	12	34		Persistent nonunion: Revised at 5 months after index surgery with RIA and additional DBM. Finally healed at 8 months after revision		
30	Double plate (4.5/3.5 dual metadiaphyseal plating (hybrid))			DBM + BMP-2	5	13				
31	Distal femur LCP (hybrid)			DBM	4	12				
32	Blade plate			DBM + BMP-2	4	16				
33	Blade plate			DBM	12	57				
34	Blade plate			DBM	5	87				
35	IMN			ICBG	17	137	CoNS			
36	IMN				5	112				
37	VA distal femur LCP (hybrid)			ICBG + DBM + FA	3	14	C. acnes			
38	Double-plate (blade plate, medial Philos plate (hybrid))			ICBG + DBM	19	13		Persistent nonunion: Revised at 6 months after index surgery with ROH (partial) and additional ICBG. Finally healed at 13 months after revision		
39	VA distal femur LCP (hybrid)			DBM	6	12				
40	Double-plate (distal femur LCP (hybrid), 3.5 pelvic reconstruction (non-locking))				36	35				
41	Double-plate (VA distal femur LCP (hybrid), medial Philos plate (hybrid))			ICBG + DBM	3	12				
42	VA-LCP			ICBG + FA	5	70				
43	Blade plate			DBM + BMP-2	10	20				
44	Double-plate (distal femur LCP (hybrid), 3.5 pelvic reconstruction (non-locking))			DBM + BMP-2	15	21				
45	FA for medial bone defect fixated with 2 screws			ICBG + DBM + FA	6	48		Femoral neck fracture not related to nonunion: ROH and replaced with partial hip arthroplasty		

AVR = aorta valve replacement; AVS = aorta valve stenosis; BPH = benign prostatic hyperplasia; CVA = cerebrovascular accident; DM = diabetes mellitus; DVT = deep vein thrombosis; HTN = hypertension; MVR = mitral valve regurgitation; PE = pulmonary embolism; RA = rheumatoid arthritis. Hu prox = humerus proximal; Hu shft = humerus shaft; Hu dist = humerus distal; Fe prox = femur proximal; Fe prox* = femur periprosthetic proximal; Fe shft = femur shaft; Fe dist = femur distal; Fe dist* = femur periprosthetic distal

AVN = Avascular necrosis; C. acnes = Cutibacterium acnes; CoNS = Coagulase-negative staphylococci; ROH = removal of hardware

**Table 3:** Patient outcome data

Outcomes	Total	Humerus	Femur
**Index procedure**			
Number of patients healed	39	15	24
Median follow-up after index in months (range)	41 (12 – 137)	34 (12 – 93)	45.5 (12 – 137)
Median time to union in months (range)	6 (2 – 37)	6 (2 – 37)	5 (2 – 36)
**Revision procedure**			
Number of patients healed	6	1	5
Median follow-up after revision in months (range)	38 (13 – 143)	69 (69 – 69)	34 (13 – 143)
Median time to union in months (range)	10.5 (4 – 42)	4 (4 – 4)	13 (5 – 42)
**Total**			
Number of patients healed after last procedure (%)	45 (100%)	16 (100%)	29 (100%)
Median follow-up after last procedure in months* (range)	41 (12 – 143)	37.5 (12 – 93)	42 (12 – 143)
Median time to union in months** (range)	6 (2 – 42)	6 (2 – 37)	6 (2 – 42)

Last procedure = index procedure or revision procedure; * = follow-up time was calculated from index procedure onwards or revision procedure onwards; ** = time to union was calculated from index procedure onwards or revision procedure onwards

### Complications and additional surgery

Six patients (13.3%) required revision surgery for persistent nonunion (Tables 2 and 3). In five (all femoral), a single revision was performed; in one (humerus shaft) a planned two-stage revision was completed. Revision surgeries took place at a median of 6 months (range 2–13) after the index surgery. The nonunion in these 6 patients ultimately healed at a median of 10.5 months (range 4–42) after the index nonunion surgery. Median follow-up after the index and revision surgery was 41 months (range 12–137) and 38 months (range 13–143), respectively. One patient developed a post-operative radial nerve palsy, which fully recovered. One patient (95-year-old female) sustained an intra-operative distal humerus fracture below the plates used for fixation of the diaphyseal humerus nonunion. The fixation was modified to include a second plate. She healed uneventfully (Fig. 2). Three patients (6.7%) requested hardware removal. One patient (2.2%) required a total hip arthroplasty for a femoral neck fracture after the distal femoral periprosthetic nonunion healed. There was one infection (2.2%) after the index procedure, which was treated with debridement, antibiotics, and placement of calcium-sulfate pellets (OSTEOSET, Wright Medical Technology Inc. Arlington, TN, USA). The infection cleared and the nonunion healed after the second stage of the revision. Four patients (8.9%) had “surprise positive cultures”. The isolated bacteria (Cutibacterium acnes and Coagulase-negative staphylococci) were treated with culture-specific antibiotics. Three of these healed without additional surgery. One patient with a proximal femoral nonunion (Coagulase-negative staphylococci detected at index procedure), required revision ORIF. She healed 5 months later.

### Literature comparison with our results

We compared our current results with literature on younger femur and humerus nonunion groups (Table 4) [20, 23, 37–63]. This table provides a comparative framework (Fig. 1). We ran a multi-variate regression analysis of Table 4 (correcting for sample size) to predict time to union based on average age and location. Data from studies published previously by us were excluded from the analyses to avoid risk for bias [23, 38, 43, 44, 53, 54, 57]. Studies of patients with a femur nonunion, revealed a longer mean healing time of 3 months (3.15 95% CI 1.5 – 4.8, *p* < 0.01) when compared with a humerus nonunion. No change occurred after adjustment for age (3.28 95% CI 1.8 – 4.7). Finally, we performed a sensitivity analysis, where we artificially assumed all patients had the maximum union time reported within each study. This did not change the findings (3.5 95% CI 1.6 – 5.4). Mean ages differed between studies. No statistical difference was identified in time to union in studies with older patients (0.07 month/per year increase in mean age (0.072 95% CI -0.002 – 0.146, *p* = 0.058)). This was also noted after adjustment for location (0.08 95% CI 0.025 – 0.135, *p* = 0.005). The sensitivity analyses, which assumed all patients with a nonunion reported maximum time to union, resulted in similar outcomes (0.08 95% CI 0.014 – 0.144, *p* = 0.018).

**Table 4:** Literature search showing union time (average, months) and age (average, years) based on nonunion location

Author	Location	Total patients (N)	Union-rate (%)	Average age (years)	Average time to union (months)
**Ring et al.** [56]	Hu proximal	25	92	61	6.0
**Walch et al. **[62]	Hu proximal	20	95	58	4.0
**Prasarn et al. **[53]	Hu proximal	19	100	70	3.5
**Badman et al. **[40]	Hu proximal	18	94	60	4.4
**Rollo et al. **[59]	Hu proximal	16	100	62	4.2
**Lin et al. **[46]	Hu shaft	86	100	42	3.6
**Marti et al. **[51]	Hu shaft	51	100	51	4
**Singh et al. **[60]	Hu shaft	40	100	36	3.7
**Donders et al. **[23]	Hu distal	62	98	48	6.8
**Rollo et al. **[58]	Hu distal	26	100	48	3.5
**Amorosa et al. **[38]	Fe proximal	46	72	60	7.1
**Lotzien et al. **[47]	Fe proximal	40	93	65	11.6
**De Vries et al. **[43]	Fe proximal	33	97	57	5.0
**Rollick et al. **[57]	Fe proximal	9	100	73	3.5
**Mardani-Kivi et al. **[50]	Fe shaft	32	100	35	5.0
**Xing et al. **[63]	Fe shaft	25	100	42	6
**Uliana et al. **[61]	Fe shaft	22	73	32	11.7
**Aslantürk et al. **[39]	Fe shaft	21	100	49	6.2
**Peng et al. **[52]	Fe shaft	21	100	47	5.3
**Maimaitiyiming et al. **[49]	Fe shaft	14	100	26	5.2
**Benazzo et al. **[41]	Fe shaft	11	100	47	6.1
**Rajasekaran et al. **[55]	Fe distal	58	98	43	7.4
**Amorosa et al. **[38]	Fe distal	32	78	56	8.4
**Gardner et al. **[44]	Fe distal	31	97	58	3.7
**Kanakeshwar et al. **[45]	Fe distal	22	100	39	6.2
**Monroy et al. **[20]	Fe distal	22	100	58	7.0
**Lu et al. **[48]	Fe distal	18	100	48	11
**Ali et al. **[37]	Fe distal	15	93	35	10.4
**Prins et al. **[54]	Fe*	19	95	71	11
**Birch et al. **[42]	Fe*	6	100	80	5.6
**Present study**	Hu	16	100	80	11.4*
**Present study**	Fe	29	100	81	9.2*

Fe = femur; Fe* = femur periprosthetic; Hu = humerus; * = average age was used for the statistical analysis and was calculated from index procedure onwards or revision procedure onwards; * = average time to union was used for the statistical analysis and was calculated from index procedure onwards or revision procedure onwards

## Discussion

In most patients, a nonunion is a disabling condition resulting in pain, reduced activity, and inability to return to pre-injury functional status [6]. The goal of nonunion surgery is to achieve a pain-free healed bone with the highest functional recovery.

Many studies have reported on techniques and complications following nonunion treatment [20, 23, 37–63]. To date, few have reported on nonunion treatment specifically in older patients [26, 64, 65]. To the best of our knowledge, no studies have restricted their findings to nonunion patients aged over 74 years.

In general, the spatiotemporal sequence of fracture healing is similar in elderly and younger populations, but the progression is protracted [66]. The cause of this is multifactorial. The expression of bone producing cells and regulation of the surrounding environment change with aging. Osteo-anabolic effects of the Wnt/BMP pathway are decreased, lowering differentiation of mesenchymal stem cells (MSC) into osteoblasts [67, 68]. Further, the functional abilities of not only MSC but also those of macrophages (important for bone healing) are impaired [5, 12]. Conversely, the pro-inflammatory cytokines such as TNF-a and Il-6 lead to increased osteoclastogenesis and increased bone resorption [67, 68].

The surrounding supportive tissue is affected by aging as well. Fracture healing requires vascularity; expression of angiogenic factors HIF-1A and VEGF has been shown to differ between young versus old animals [69]. Further, the endothelial precursor cells (EPC) required for new blood vessel formation are compromised [68]. Additionally in females specifically, the postmenopausal estrogen deficiency negatively affects bone healing response [70]. It is well accepted that density of bone decreases with age, but recent studies showed osteoporosis and decreased bone density are not risk factors for nonunion when matched for age and sex [71, 72]. These findings are confirmed in a large retrospective nonunion study [7]. The cause of nonunion in patients with osteoporosis is likely multifactorial and includes age-related changes in bones as well as challenges in achieving stable internal fixation [73].

The exact incidence of nonunion in older patients is disputed, but given the macro trend of increased aging, they will continue to occur. An examination of > 47,000 Medicare patients aged > 75 years showed a lower rate of nonunion compared to those < 70 [19]. Another study by Mills et al. showed not only that the total number of nonunion in this age category of patients is lower than previously thought, but also that the in the 75–85 year age bracket was 1.2% versus 3% in the younger group (25–44 years) [74].

Concerns in the very elderly include poor bone stock and co-morbidities jeopardizing surgical outcomes. Fortunately, our results and those of others demonstrate good and reproducible outcomes of operative treatment for elderly nonunion patients [25].

Similar to our younger nonunion patients, we utilized a protocol of debridement, reduction, stable fixation, bone grafting, and early motion [23, 38, 43, 44, 51, 53, 54, 56].

Few clinical reports focus on nonunion in seniors. Zura et al. performed a prospective cohort study of 2.5 million United States Medicare patients. Of these, 47,437 had a fracture or nonunion. Patients with a fracture but without a nonunion were older (75 years) versus those with a nonunion (69.2 years) [19]. Taormina et al. also published a nonunion study, but reported on a younger nonunion population than ours (64 years or older) [64]. In their work, which focused on nonunions of forearm, femur, humerus, clavicle, tibia, foot and ankle, they found patients age was not associated with achieving union and, importantly, that advanced age was not associated with worse outcomes.

Finally, Tanner et al. reported a matched pair analysis on outcome of lower limb nonunion treatment in patients younger or older than 60 years [26, 65]. Treatment was according to the diamond concept. Advanced age did not negatively influence outcome of nonunion surgery [65].

This study has limitations. Although these patients are a prospectively documented cohort in our orthopedic trauma database, pre-operative or post-operative patient-based scores were unavailable. Therefore, clinical improvement after operative treatment could not be assessed. However, osseous union is a logical endpoint after nonunion treatment and it is well known that clinical and radiological outcome correlate regardless of patient age [65]. Additionally, since all three orthopedic surgeons are experienced nonunion surgeons, the results may not be easily reproducible in a general orthopedic setting.

Also, a large group of 30 patients (14 humeral nonunions and 16 femoral nonunions) were excluded because they had shorter than 12 months follow-up due to large traveling distance, concomitant disease, or death. This may have led to selection bias. We included the Weber and Çech classification in this series as it is a part of the NUSS scoring system and many surgeons still refer to it in practice. In reality, we feel the Weber and Çech system likely has little applicability to modern nonunion series as most patients (80% in this series) have already undergone ORIF and are highly unlikely to show the classic historical patterns of atrophic and hypertrophic nonunions seen when many long bone fractures were treated nonoperatively. We have done our best to classify all of the patients into one of the 3 groups, however, nonunions after ORIF usually display some level of callus formation on plain film and CT-scans, and the difference amongst hyper, oligo, and atrophic is quite subjective.

Furthermore, use of bone grafting was not standardized and amount of graft in relation to defect size was unknown. Comparing contributions of BMP versus DBM versus allograft and/or autologous bone graft was not part of the study. Autologous bone graft continues to be the gold standard for bone grafting [75]. Lastly, the reviewed literature did not have a clear-cut definition for nonunion so comparing our results with existing studies was difficult.

The series has several notable strengths. All patients were treated using a standardized surgical protocol consisting of debridement, reduction, and stable fixation., inclusion of the NUSS, radiographic evaluation by a musculoskeletal radiologist, and > 12 months follow-up. Another benefit of the study is inclusion of the NUSS.

Rather than reporting a mixed cohort of nonunion locations, we limited our study to the two most frequent occurring nonunion (humerus and femur) in the elderly. While others reported on nonunion in the elderly, their minimum inclusion age was much lower than ours [26, 64, 65]. We believe our group represents a different age bracket. Lastly, it represents two geographically diverse groups with very different health care systems (European and American respectively) showing equal outcomes. This suggests that these results are reproducible in different health care systems.

As we look to the future, potential treatment opportunities to accelerate fracture and nonunion healing may be different for the elderly as the native MSC and endothelial precursor cells needed for new blood vessel formation are compromised in number and/or function. Further options may involve bone anabolic pharmaceuticals (parathormone, growth factors such as BMP), reduction of senescent progenitor cells, or decreasing the *inflamm-aging* state in the elderly with a low-grade, non-steroidal anti-inflammatory drugs (NSAID). [13, 68] Despite enormous progress and promising results of using these adjuncts in animal studies, clinical success has been lacking. This should be kept in mind when using these adjuncts, as this is a vulnerable group of patients that attracts a highly competitive bone regeneration industry that is often based on unsupported claims of success [24].

## Conclusions

Following a standardized protocol of aggressive debridement, anatomic alignment, stable fixation with compression, liberal use of bone grafting, and early motion was found to reliably achieve bony union in the elderly nonunion patient.

Given these results, we recommend nonunion surgery in the geriatric patient population after thorough risk assessment and preoperative medical and surgical optimization is achieved. Advanced patient age should not be a contra-indication for nonunion surgery.

## Data Availability

The datasets used and/or analyzed during the current study are available from the corresponding author on reasonable request.

## Abbreviation

MSC: Mesenchymal stem cells
ORIF: open reduction and internal fixation
NUSS: Non-Union Scoring System
ICBG: iliac crest bone graft
DBM: demineralized bone matrix
VA-LCP: variable angle locking plates
FA: fibula allograft
MSC: mesenchymal stem cells
EPC: endothelial precursor cells
NSAID: non-steroidal anti-inflammatory drugs
